# Intra-amniotic transplantation of brain-derived neurotrophic factor-modified mesenchymal stem cells treatment for rat fetuses with spina bifida aperta

**DOI:** 10.1186/s13287-022-03105-6

**Published:** 2022-08-13

**Authors:** Wei Ma, Xiaowei Wei, Hui Gu, Dan Liu, Wenting Luo, Songying Cao, Shanshan Jia, Yiwen He, Lizhu Chen, Yuzuo Bai, Zhengwei Yuan

**Affiliations:** 1 Key Laboratory of Health Ministry for Congenital Malformation, Department of Pediatric Surgery, Shengjing Hospital, China Medical University, No. 36, Sanhao Street, Heping District, Shenyang, 110004 China; 2 Department of Ultrasound, Shengjing Hospital, China Medical University, Shenyang, China; 3 Department of Pediatric Surgery, Shengjing Hospital, China Medical University, Shenyang, China

**Keywords:** Spina bifida aperta, Mesenchymal stem cells derived from bone marrow, Brain-derived neurotrophic factor, Intra-amniotic transplantation, Prenatal treatment

## Abstract

**Background:**

Spina bifida aperta (SBA) is a relatively common clinical type of neural tube defect. Although prenatal fetal surgery has been proven to be an effective treatment for SBA, the recovery of neurological function remains unsatisfactory due to neuron deficiencies. Our previous results demonstrated that intra-amniotic transplanted bone marrow mesenchymal stem cells (BMSCs) could preserve neural function through lesion-specific engraftment and regeneration. To further optimize the role of BMSCs and improve the environment of defective spinal cords so as to make it more conducive to nerve repair, the intra-amniotic transplanted BMSCs were modified with brain-derived neurotrophic factor (BDNF-BMSCs), and the therapeutic potential of BDNF-BMSCs was verified in this study.

**Methods:**

BMSCs were modified by adenovirus encoding a green fluorescent protein and brain-derived neurotrophic factor (Ad-GFP-BDNF) in vitro and then transplanted into the amniotic cavity of rat fetuses with spina bifida aperta which were induced by all-trans-retinoic acid on embryonic day 15. Immunofluorescence, western blot and real-time quantitative PCR were used to detect the expression of different neuron markers and apoptosis-related genes in the defective spinal cords. Lesion areas of the rat fetuses with spina bifida aperta were measured on embryonic day 20. The microenvironment changes after intra-amniotic BDNF-BMSCs transplantation were investigated by a protein array with 90 cytokines.

**Results:**

We found that BDNF-BMSCs sustained the characteristic of directional migration, engrafted at the SBA lesion area, increased the expression of BDNF in the defective spinal cords, alleviated the apoptosis of spinal cord cells, differentiated into neurons and skin-like cells, reduced the area of skin lesions, and improved the amniotic fluid microenvironment. Moreover, the BDNF-modified BMSCs showed a better effect than pure BMSCs on the inhibition of apoptosis and promotion of neural differentiation.

**Conclusion:**

These findings collectively indicate that intra-amniotic transplanted BDNF-BMSCs have an advantage of promoting the recovery of defective neural tissue of SBA fetuses.

**Supplementary Information:**

The online version contains supplementary material available at 10.1186/s13287-022-03105-6.

## Background

Spina bifida aperta (SBA) is a congenital anomaly caused by neural tube dysraphism, in which neural tissue communicates with the external environment. Complete early closure surgery is always accomplished within 72 h of birth to prevent significant neurological decline and minimize the risk of infection or injury from any exposure defect. Although this intervention can stabilize the condition at birth, it does not prevent neurological damage, as lower limb function usually deteriorates during pregnancy [1]. In the last 10 years, in utero prenatal SBA repair has been performed by some highly specialized centers. Prenatal surgery was proven to reduce the impact of intrauterine injury in an SBA lamb model [2–4] and improve the functional outcome by the Management of Myelomeningocele Study randomized controlled trial [5, 6]. However, in utero repair can be implemented only in a limited selection of patients, including in fetuses with a lesion from the T1-Sacrum, an Arnold Chiari malformation, and age 19–25 weeks, to prevent future complications [1, 7]. Therefore, irrespective of whether they are performed in the prenatal or postnatal period, these surgical repair methods are adopted after neuronal injury. We speculate that this may explain the neurodevelopmental impairment observed in children, as well as why the recovery of neurological function remains unsatisfactory [8]. Perhaps, earlier treatment will achieve better results before a large number of neurons are damaged.

Over-apoptosis of the neuroepithelium is an important mechanism during SBA development. We have previously observed that the increase in apoptosis and the decrease in proliferation induce deficiencies of sensory and motor neurons in the defective neural tube [9–12]. Therefore, we consider that the unsatisfactory functional outcomes of postnatal and prenatal repair of defective neural tubes might be due to the deficiency of functional neurons. Although repair of the lesions could avoid further aggravation of injury, neural functional recovery is insufficient.

We previously attempted to perform intra-amniotic delivery of a recombinant adenovirus encoding brain-derived neurotrophic factor (BDNF) to promote neural recovery, and our results showed that BDNF was increased significantly and demonstrated neurotrophic effects in defective spinal cords [13]. BDNF is widely recognized as an important neurotrophin and plays a key role in the development of the nervous system by affecting cell differentiation, neuronal development, neurogenesis, synaptogenesis, and synaptic plasticity [13–16]. This delivery method could overcome the limitation of a short in vivo half-life of the neurotrophin to some extent and rapidly and sharply increase the expression of BDNF in SBA spinal cords improving apoptosis and promoting neuronal regeneration. However, the infection of adenovirus is not absolutely specific to defective nerve tissue. Adenovirally expressed BDNF could gather on the defective spinal cords and also express in eyelids, lips, limb buds and other tissues [13]. The clinical applicability of such gene delivery is still limited due to vector safety, unpredictable side effects, and complex ethical issues. We have also performed intra-amniotic bone marrow mesenchymal stem cell (BMSC) therapy, based on various positive qualities of BMSC, including their convenient isolation, strong self-renewal ability, multi-lineage differentiation, selective migration to damaged tissues, and secretion of various trophic factors that develop a regenerative microenvironment [17]. Following intra-amniotic BMSC transplantation in SBA rat models, we found that BMSCs spontaneously migrated to the SBA lesion, covered the defective tissues, promoted repair of nerves and the epidermis, and improved neural function [18]. Considering that BMSCs can be easily modified to improve therapeutic efficacy [19], to further optimize the treatment of SBA by intra-amniotic stem cell transplantation combined with the advantages of gene therapy, we used a recombinant adenovirus (Ad) encoding BDNF to transfect the BMSCs before transplantation in the present study.

We speculated that if intra-amniotically transplanted BMSCs carried BDNF, the BDNF level could be elevated by lesion-specific engrafted BMSCs in SBA spinal cords. This would further optimize the role of BMSCs and make the environment in defective spinal cords more conducive to nerve repair. To test this hypothesis, BMSCs were modified with BDNF before intra-amniotic transplantation, and the therapeutic potential of BMSCs-BDNF for SBA rat fetuses was evaluated.

## Materials and methods

### Experimental animals

Outbred Wistar rats, aged 10–12 weeks (250–300 g) and 4 weeks (approx. 100 g), were purchased from the Animal Center of China Medical University. All rats were provided food and water ad libitum and were housed under pathogen-free conditions with a 12-h light/dark cycle. Adult mice were allowed to mate freely at night. The morning in which the vaginal plugs or the sperms on the vaginal smears were observed was considered day 0 of embryonic development (E0). At E10, rat embryos were induced to develop SBA by a single intra-gastric gavage of all-trans-retinoic acid (atRA; 4% wt/vol in olive oil; 140 mg/kg body weight) in pregnant rats [20]. All procedures were performed in accordance with National Institutes of Health guidelines for the care and use of laboratory animals and approved by the Committee for Animal Care at Shengjing Hospital of China Medical University.

### Isolation, culture, and transfection of BMSCs

BMSCs were isolated from the femurs of wistar rats aged 4 weeks, then identified phenotypes by flow cytometry using specific antibodies (CD90, CD44, CD73, CD29, CD34 and CD45), and analyzed differentiation potential, as previously reported [21]. Briefly, BMSCs were cultured in DMEM/F12 (HyClone) supplemented with 10% fetal bovine serum (FBS; Gibco) in a 25 cm^2^ tissue culture flask at 37 °C and 5% CO_2_. The culture medium was replaced every 1–2 days. The primary culture (P0) was cultured until approximately 80% confluency, following which the cells were subcultured with 0.25% trypsin and 0.5 mM ETDA (Invitrogen) to further expand the cultivation. After subculture for 3–5 passages, the cells were prepared for transplantation experiments.

The coding region sequences of green fluorescent protein (GFP) and BDNF were cloned into E1- and E3-deleted human adenovirus type 5 vectors under the control of a cytomegalovirus (CMV) promoter to generate the recombinant adenovirus (i.e., Ad-GFP-BDNF) and GFP expressing adenoviral vector (i.e., Ad-GFP). Next, 5 μL adeno-5 vector (100 pfu/cell; SinoGenoMax Co., Ltd., China) was added to the culture medium 24 h before transplantation to label the BMSC-derived cells. The transfected BMSCs were collected, washed, and resuspended in phosphate-buffered saline (PBS) before transplantation.

### Intra-amniotic BDNF-BMSC transplantation

Intra-amniotic BDNF-BMSCs, BMSCs or PBS injection was performed on E15. First, pregnant rats were anesthetized with pentobarbitone sodium (40 mg/kg body weight). Then, an incision was made in the abdominal wall to expose the uterus. Under the operation microscope, the embryos with SBA were randomly divided into three groups (*n* = 30 per group), including the PBS-injected group, the BMSC-injected group, and the BDNF-BMSC-injected group. Next, 2 μL of cell suspension (approx. 5 × 10^6^ cells) or an equal volume of PBS was injected into the amniotic fluid through the uterine wall with a micropipette, and the locations of the injected fetuses were recorded. Finally, the uterus was gently returned to the abdomen before closing the abdominal wall. The pregnant rats were kept warm after the operation until they recovered from anesthesia and returned to the feeding cage. On E20, the female rats were euthanized with excessive pentobarbital sodium, and the injected fetuses were harvested for further analysis.

### Real-time polymerase chain reaction

The total RNA of the spinal cords was extracted by the TRIzol Reagent kit (Invitrogen) according to the manufacturer’s instructions. cDNA was prepared using the PrimeScript reagent kit (Takara, Japan) for qRT–PCR analysis by SYBR Premix Ex Taq (Tli RNaseH Plus; Takara) in the Applied Biosystems 7500 (Applied Biosystems, Foster City, CA, USA). Primer Premier software (version 5.0) was used to design the real-time polymerase chain reaction (RT-PCR) primers. The 2^−ΔΔCt^ method was used to calculate the relative expression after normalization to GAPDH. The primer sequences are given in Table 1.

**Table 1:** Sequences of primers used in this study

Gene	Accession number	Primer sequences (5’-3’)/exon location (nt)	Annealing Tm (°C)	PCR product (bp)
BDNF	NM_001270630.1	Sense: ATGGTTATTTCATACTTCGGTTGC/391-414 Antisense: CTCAAAAGTGTCAGCCAGGGA/567-547	60	177
BCL2	NM_016993.1	Sense: ACGAGTGGGATACTGGAGATGAAGACT/317-343 Antisense:ACGTCCTGGCAGCCGTGTCT/442-423	61	126
BAX	NM_017059.2	Sense: TGGAAGAAGATGGGCTGAGGC/651-671 Antisense: CATTCCCACCCCTCCCAATAAT/789-768	60	139
CASP3	NM_012922.2	Sense: GGAACGAACGGACCTGTGG/441-459 Antisense: CGGGTGCGGTAGAGTAAGC/660-642	60	220
BRN3A	XM_001076964	Sense: ATCGCGGTGTCCCAGGGCAAGA/196-218 Antisense: CGAGATGTGGTCCAGCAGGTCA/363-341	60	168
ISLET1	NM_017339.3	Sense:CATCGAATGTTTCCGCTGTG/308-327 Antisense:GGTCTTCTCGGGCTGTTTGT/548-529	60	241
SYN	NM_006950.3	Sense:TGGGCAAGGTCAAGGTAGA/959-977 Antisense:TGGACACGCACATCGTATTTA/1079-1059	60	121
SYT	NM_005639.2	Sense: CGCTGAGAAAGAAGAGCAAGA/1452-1472 Antisense:ATAAGCCACCCACATCCATC/1582-1563	60	131
GAPDH	NM_017008.4	Sense: TGCCGCCTGGAGAAACCTGC/808-827 Antisense: AGCAATGCCAGCCCCAGCAT/975-956	60	168

### Fluorescence imaging and tissue preparation

On E20, the injected fetuses were harvested, and a fluorescence stereomicroscope (M165FC, Leica, Germany) fitted with a Nikon DS-Qi2 digital camera (NY-1S35, Nikon, Japan) was used to take the back images of the fetuses. The skin lesion area of these fetuses was measured using BR analysis software. Then, the fetuses were perfused transcardially with 15 mL physiological saline, followed by 25 mL 4% paraformaldehyde. The lumbosacral spinal column—containing muscle, spinal cord, and subcutaneous tissue—was dissected from the fetuses and maintained overnight in 4% paraformaldehyde. The tissues were then dehydrated in 20% sucrose for 24 h and then embedded in the optimal cutting temperature compound (OCT, SAKURA, Japan). Serial 30-μm-thick slices from the lumbosacral spinal column were prepared using a freezing microtome (Microm HM525, Thermo, Germany) before attaching to polylysine-coated glass slides. The GFP-positive cells on the slides were observed and counted by fluorescence microscopy (80i, Nikon). All sections were marked and preserved at − 80 °C for TdT-mediated dUTP Nick-End Labeling (TUNEL) and immunofluorescence staining.

### TUNEL and immunofluorescence staining

TUNEL and GFP immunofluorescence staining were performed simultaneously using the In Situ Cell Death Detection Kit (Roche) and mouse anti-GFP antibody. According to the manufacturer’s protocol, the sections were first blocked with PBS containing 10% FBS and 0.1% Triton x-100 after antigen retrieval, before incubating with a TUNEL reaction mixture at 37 °C for 30 min in the dark. Then, mouse anti-GFP antibody (1:200, AG271, Beyotime Institute of Biotechnology, China) and TRITC-conjugated goat anti-mouse (1:100; AP124R, Millipore) were added and incubated after washing. The sections were incubated for 5 min with DAPI (C1002; Beyotime Institute of Biotechnology), before being mounted with anti-fade mounting medium and observed under a C1 confocal microscope (Nikon). The red and green channels were interchanged such that the TUNEL-positive cells were red and the GFP-positive cells were green. Fields of view (40 × magnification) and representative images were captured for apoptotic cell counts.

For immunofluorescence staining, the primary antibodies we used were mouse anti-BRN3A (1:50; MAB1585, Millipore, Germany), K19 (1:100; sc-376126; Santa, China), SYNAPTOTAGMIN (SYT, 1:200; MAB5200, Millipore), rabbit anti-GFP (1:200; AG279; Beyotime Institute of Biotechnology, China), ISLET1 (1:200; ab20670, Abcam, Britain), and SYNAPSIN1 (SYN, 1:200; AB1543P, Millipore), and the secondary antibodies included Alexa Fluor 488-conjugated goat anti-rabbit IgG antibody (1:100; AB_2539798, Invitrogen), TRITC-conjugated goat anti-mouse IgG antibody (1:100; AP124R, Millipore), Alexa Fluor 488-conjugated goat anti-mouse IgG antibody (1:100; AB_2534088, Invitrogen), and TRITC-conjugated goat anti-rabbit IgG antibody (1:100; AP187R, Millipore). All sections were counterstained with DAPI. The immunofluorescence analysis was performed according to standard procedures. Representative images were obtained using a C1 confocal microscope (Nikon). The differentiation rate of engrafted BDNF-BMSCs was determined as the number of a specific marker and GFP double-positive cells/total number of GFP-positive cells/field.

### Automatic western blot analysis

Protein expression analysis was performed using a Jess automated system (ProteinSimple, Santa Clara, CA, USA) according to the ProteinSimple user manual. In brief, tissue lysate samples were mixed into a master mix (ProteinSimple) containing sample buffer and DTT to the final concentration and then heated at 95 °C for 5 min. The samples, blocking reagent, primary antibodies, HRP- or NIR-conjugated secondary antibodies, luminol–peroxide mix, and wash buffer were added to designated wells in a 384-well plate. After plate loading, the separation electrophoresis and immunodetection steps proceeded in the capillary system and were fully automated in the Jess automated system. The digital images were analyzed with Compass software (ProteinSimple), and the quantified data of the detected proteins were reported as molecular weight and signal/peak intensity. The target protein antibodies were BDNF (1:25; NB100-98,682, NOVUS), BCL2 (1:25; SC-7382, NOVUS), BAX (1:50; D2E11, Cell Signaling), CASP3 (1:25; NB100-23,708, NOVUS), BRN3A (1:50; MAB1585, Millipore), ISLET1 (1:50; NBP2-14,999, NOVUS), SYNAPSIN1 (1:25; AB1543P, Millipore), SYNAPTOTAGMIN (1:25; MAB5200, Millipore), and GAPDH (1:100; 60,004-1-Ig, Proteintech). For quantitative data analysis, all of the target signals were normalized against GAPDH as a loading control.

### Protein microassay analysis for amniotic fluid

BMSC- and BMSC-BDNF-injected amniotic fluid were extracted from E20 fetuses. The RayBio Biotin Label-Based Rat Antibody Array 1 (RayBiotech) was used to determine 90 cytokines in the amniotic fluid according to the manufacturer’s instructions. Briefly, the primary amines of the proteins were biotinylated, the glass slide arrays were blocked, and the biotin-labeled samples were added to the slide, which was preprinted with capture antibodies, and incubated to allow the target proteins to interact. Streptavidin-conjugated fluorescent dye (Cy3 equivalent) was then applied to the array. Finally, the glass slide was dried and the signals were visualized by laser fluorescence scanning. Differentially expressed proteins were defined as fold change > 3 or < 0.3 (fold change = BDNF-BMSC-injected group/BMSC-injected group) and *p* < 0.05. STRING (Search Tool for the Retrieval of Interacting Genes/Proteins, https://string-db.org/cgi/input.pl) was used for protein–protein interaction and biological process analysis of differentially expressed cytokines.

### Statistical analysis

All data are presented as the mean ± standard error of the mean and were analyzed using SPSS version 16.0 (SPSS, Inc., Chicago, IL, USA). All analyses were performed in a double-blind manner and duplicated one to two times, and representative experiments are shown. A one-tailed Student’s *t* test was used for comparisons between two groups, and for comparisons between multiple groups, one-way ANOVA was used. *p* values < 0.05 were considered statistically significant.

## Results

### Expression of BDNF in rat spinal cords with SBA was decreased on different embryonic days

**Fig. 1:**
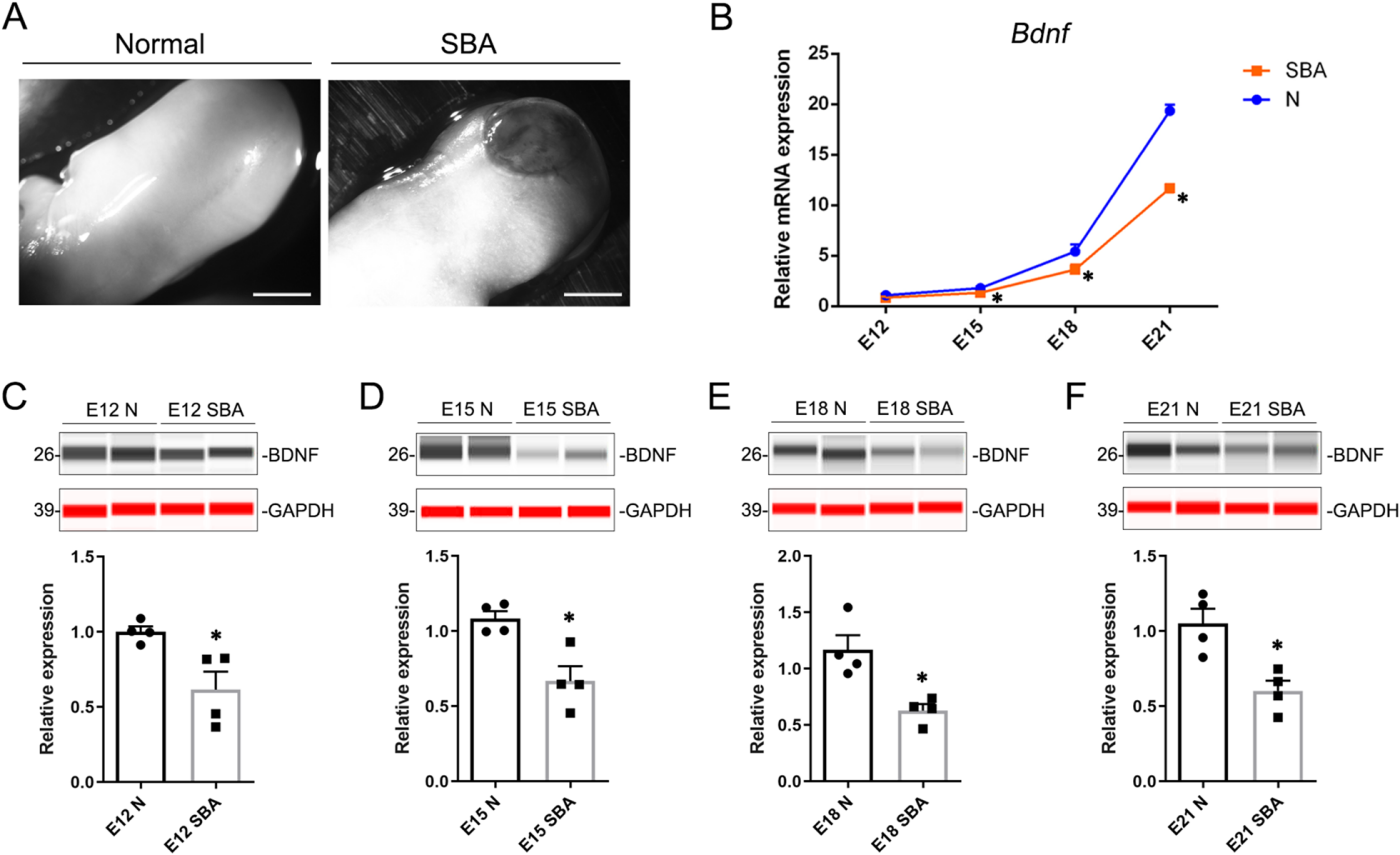
Expression pattern of BDNF in the spinal cords of normal and SBA rat fetuses. **A**. A typical back photograph from a normal fetus and an SBA fetus induced by atRA. **B**. Expression-level analysis of BDNF mRNA in normal and SBA spinal cords on E12, E15, E18, and E21. **C**–**F**. Immunodetection of BDNF protein in the spinal cords of normal and SBA fetuses at E12, E15, E18, and E21 by simple western shows a significant reduction in BDNF protein in the SBA spinal cords. Gray–white stripes were detected by the HRP channel; red stripes were detected by the NIR channel. The stripes and quantification of relative protein levels were determined from peak areas detected by a size-based capillary electrophoresis instrument. Abbreviations: N: normal fetal rat spinal cord, SBA: spina bifida aperta, E: embryonic days, *p < 0.05

The atRA-induced SBA rat model showed obvious skin lesions and exposed defective spinal cord following cesarean section on embryonic day (E) 20 (Fig. 1A). The spinal cords from normal and SBA fetal rats on E12, E15, E18, and E21 were collected for RNA and protein extraction. The results of RT-qPCR and western blotting showed that the BDNF mRNA expression increased gradually with embryonic age (Fig. 1B). Moreover, both the mRNA and protein level of BDNF in the SBA spinal cord was significantly downregulated compared to normal embryos/fetuses of the same embryonic age at E12, E15, E18, and E21, during the period from teratogenesis to birth (Fig. 1B–F). The diminished level of BDNF might be detrimental to the neural development of fetal rats with SBA.

### BDNF expression increased in defective spinal cords, and the skin lesions decreased after intra-amniotic BDNF-BMSC transplantation

**Fig. 2:**
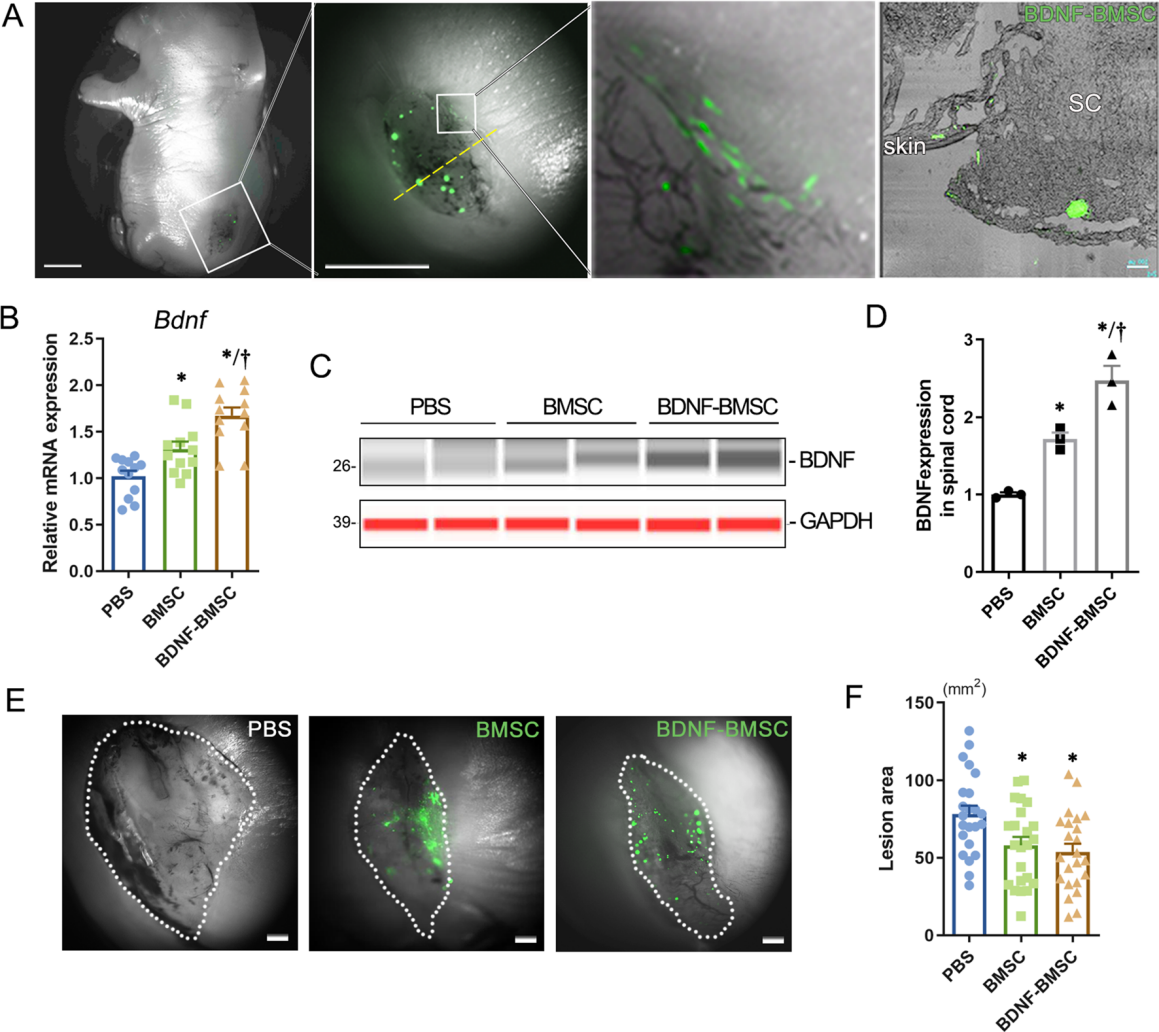
Intra-amniotic transplanted BDNF-BMSCs implanted into defective spinal cord and skin. **A**. Typical images observed under a stereoscopic fluorescence microscope; the white box is magnified, and the scale: 2.5 mm; the yellow dotted line indicates the location of the rightmost frozen section which shows that the transplanted bone marrow mesenchymal stem cells (BMSCs) engrafted into the defective spinal cord and the edge of the broken skin, scale: 100 μm. **B**: Quantitative analysis of the relative mRNA expression of BDNF in SBA spinal cords of PBS-injected, BMSC-injected and BDNF-BMSC-injected fetuses (*n* = 12 per group). **C**: The protein expression of BDNF and GAPDH in the defective spinal cord was detected by the simple western system in PBS-, BMSC-, and BDNF-BMSC-injected groups. **D**. Quantification of relative protein levels of BDNF in different transplantation groups from Figure C. *Compared to the PBS-injected group, *p* < 0.05; †Compared to the BMSC-injected group, *p* < 0.05. **E**. Representative images of E20 rat fetus with SBA after PBS injection, BMSC transplantation, and BDNF-BMSC transplantation taken by fluorescent stereomicroscopy; the transplanted cells were labeled by GFP. The white dots indicate the edge of the defective skin lesions. **F**. Quantitative analysis of the area of skin lesions in the BDNF-BMSC-injected, BMSC-injected, and PBS-injected groups (*n* = 23 per group). *Compare to the PBS-injected group, *p* < 0.05

Before intra-amniotic transplantation, the cells to be transplanted were transfected by Ad-GFP-BDNF, GFP as a reliable indicator in that it persisted until the fetuses were harvested. Successfully engrafted BDNF-BMSCs could be directly observed under a fluorescent stereoscopic microscope on E20. The location of the GFP expression indicated that the BDNF-BMSCs tended to engraft on the dorsal defective spinal cord and skin. The engrafted cells were scattered (Fig. 2A). Through freezing serial sectioning, we also observed that the transplanted BMSCs engrafted into the defective spinal cord and the edge of the broken skin (Fig. 2A), suggesting that these engrafted BMSCs-BDNF have the opportunity to participate in the repair of the defective nerve and skin. RT-qPCR and western blot results showed that the expression of BDNF at both the mRNA and protein level was significantly upregulated in the BDNF-BMSC-injected group compared to the PBS- or BMSC-injected group, respectively (Fig. 2B–D). By measuring the lesion area, we found that the area of the skin lesions in the back of SBA rat fetuses transplanted with BMSCs or BMSCs-BDNF was significantly smaller than those in the non-transplantation group (Fig. 2E, F).

### The apoptosis in SBA spinal cords was alleviated by intra-amniotic BDNF-BMSC transplantation

**Fig. 3:**
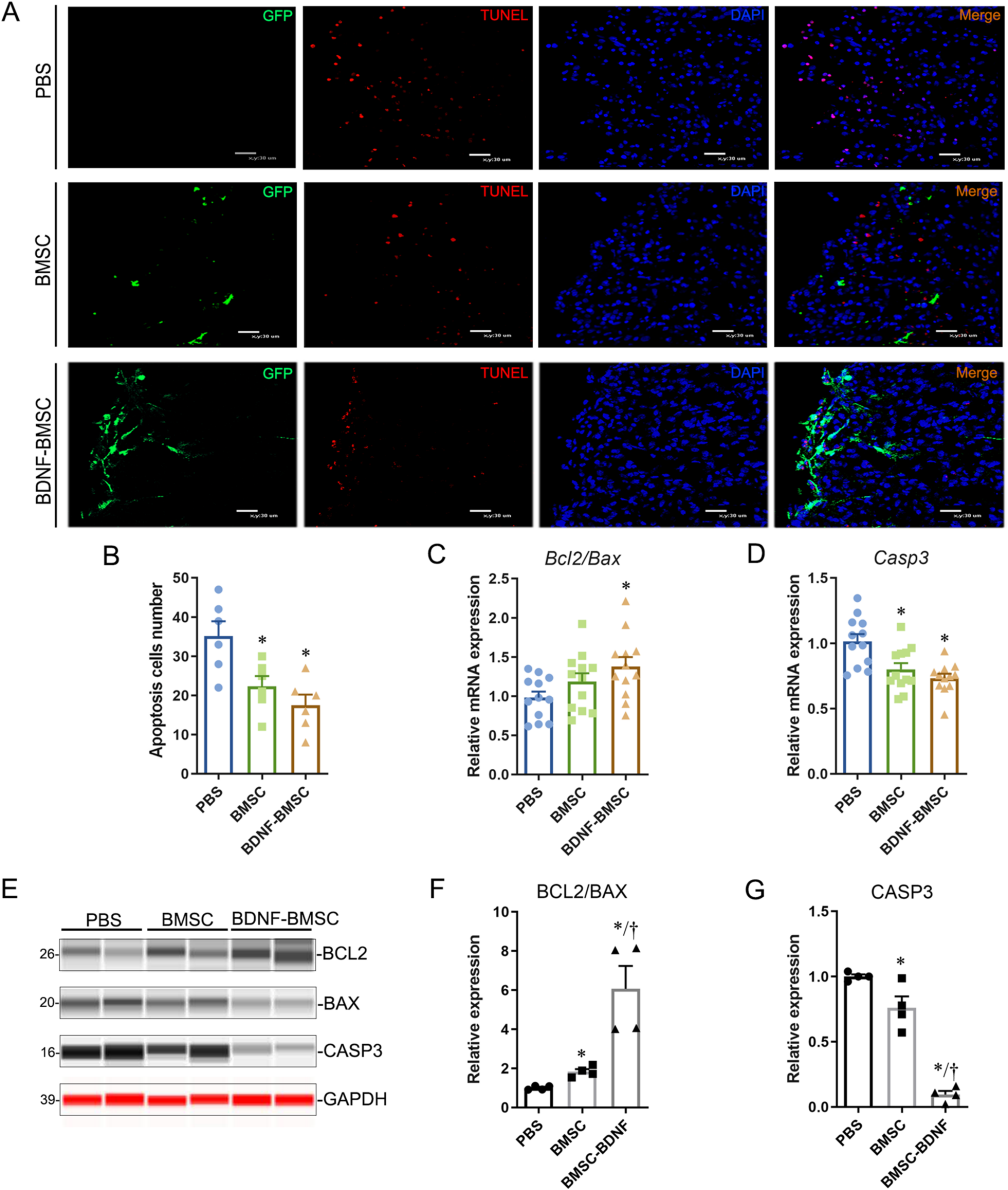
Engrafted BDNF-BMSC significantly reduced the apoptosis in defective spinal cords. **A**. Typical images of double staining of TUNEL and GFP antibody in the spinal dorsal horn were observed in the BDNF-BMSC-injected group, the BMSC-injected group, and the PBS-injected group under a fluorescence microscope. Scale bar: 30 μm. **B**. Quantification of apoptotic cells was performed under 40 × field (*n* = 6/group). The apoptotic cells in the dorsal spinal cord of the BDNF-BMSC-injected group and BMSC-injected group were significantly decreased compared to the PBS injection group. **C**–**D**. Quantitative analysis of the relative mRNA expression of BCL2, BAX, and CASP3 in the spinal cords of PBS-injected, BMSC-injected, and BDNF-BMSC-injected fetuses (*n* = 12/group). *The difference was significant compared to the PBS group (*p* < 0.05). **E**. Simple western detection of BCL2, BAX, and CASP3 protein expression in the spinal cords from SBA fetuses after intra-amniotic injection of PBS, BMSCs, and BDNF-BMSCs. Gray–white stripes were detected by the HRP channel; red stripes were detected by the NIR channel. **F**–**G**. Quantification of the relative protein levels determined from the special peak area of BCL2, BAX, and CASP3 shown in E. *Significant difference compared to the PBS-injected group, †Significant difference compared to the BMSC-transplanted group, *p* < 0.05

Excessive apoptosis of neuroepithelial cells is considered an important cause of neural tube malformation [22]. The dorsal horn of the SBA spinal cord is exposed to amniotic fluid, which is the area with the highest rates of apoptosis. To investigate whether intra-amniotic BDNF-BMSCs delivery could reduce apoptosis in the dorsal horn of the SBA spinal cord, serial frozen sections of defective spinal cords were prepared for double staining with TUNEL and GFP. The results showed that the number of apoptotic cells was significantly reduced in BMSC- and BDNF-BMSC-engrafted spinal cords compared to the controls and slightly decreased in the BMSC-BDNF group compared to the BMSC group (Fig. 3A, B). The relative mRNA levels of BCL2, BAX, and Caspase-3 (CASP3) in the defective spinal cords were detected by RT-PCR. We found that the BCL2/BAX ratio was increased in the BDNF-BMSC-injected group compared to the PBS-injected group. Moreover, CASP3 was significantly decreased in the BDNF-BMSC-injected group compared to the PBS-injected group and slightly decreased compared to the pure BMSC transplantation group (Fig. 3C, D). The protein levels of these apoptosis-related genes were detected by the simple western system. The results showed in the BDNF-BMSC-injected group, BCL2/BAX in defective spinal cords was significantly upregulated, while CASP3 was significantly downregulated compared to BMSC- and PBS-injected groups, respectively (Fig. 3E–G). These results indicate that intra-amniotic BDNF-BMSC transplantation could reduce apoptosis in SBA spinal cords and it is better than BMSCs in this regard.

### The repair of neurons was promoted in defective spinal cords

**Fig. 4:**
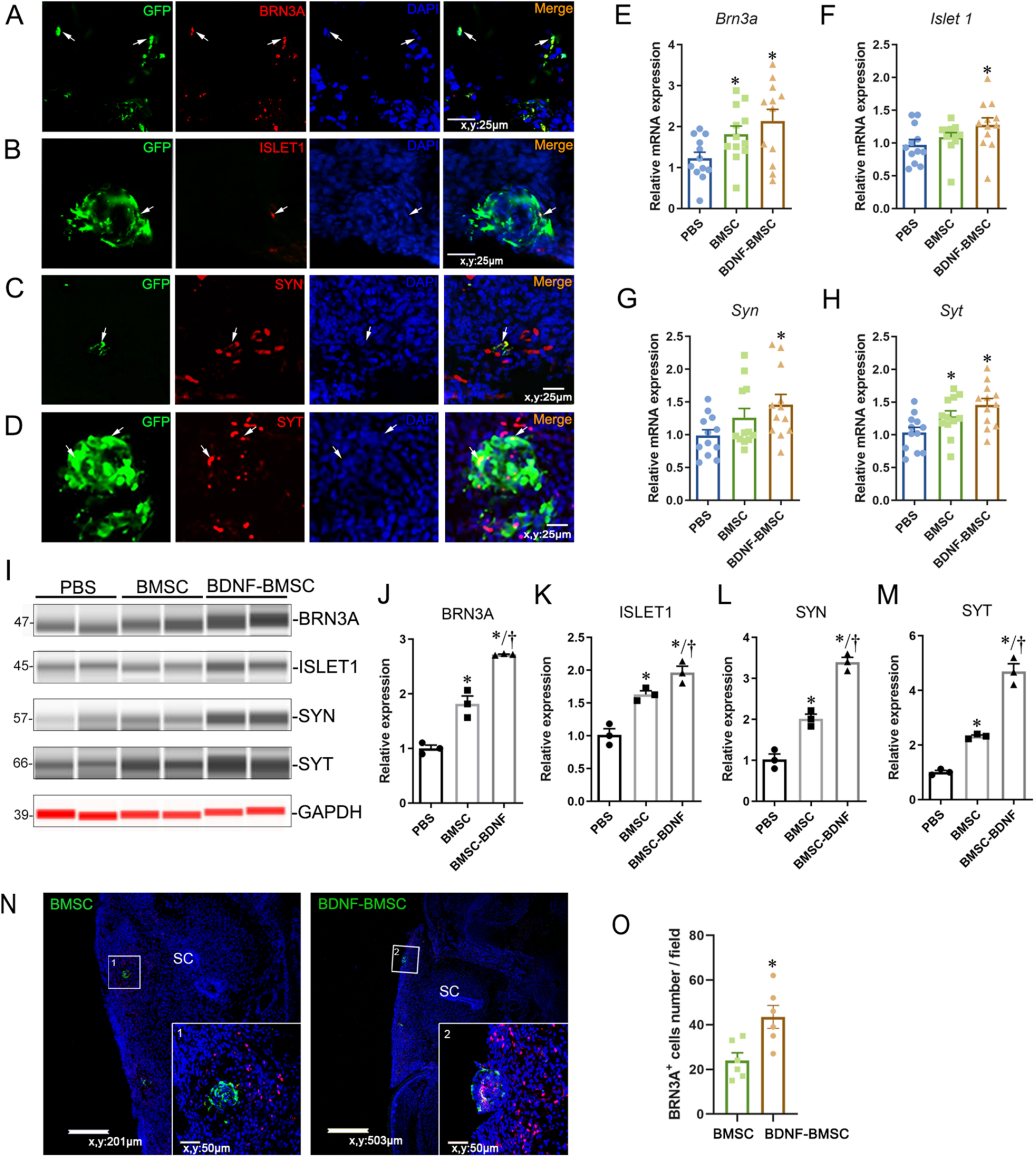
Engrafted BDNF-BMSCs expressed neuron-related specific markers in the spinal cord of SBA. **A**–**D**. Double fluorescent staining of BRN3A, ISLET1, SYT, and SYN with GFP in the sections of defective spinal cords with BDNF-BMSCs engraftment. Typical double-positive cells are labeled with arrows. **E**–**H**. The relative mRNA expression of BRN3A, ISLET1, SYN, and SYT in the spinal cords of BDNF-BMSC-, BMSC-, and PBS-injected groups was quantitatively analyzed by RT-qPCR (*n* = 12/group). **I**. Simple western system detection of BRN3A, ISLET1, SYN, and SYT protein in the spinal cords from SBA fetuses after intra-amniotic injection of PBS, BMSCs, and BDNF-BMSCs. Gray–white stripes were detected by the HRP channel, and red stripes were detected the by NIR channel. **J**–**M**. Quantification of relative protein levels determined from the special peak area of BRN3A, ISLET1, SYN, and SYT shown in I. **N**. Representative images of GFP and BRN3A double staining in defective spinal cords with BDNF-BMSCs and pure BMSC engraftment. The images in the small white box are enlarged in the lower right corner. SC: spinal cord. **O**. The BRN3A^+^ cells around the engrafted BMSCs-BDNF or BMSCs in defective spinal cords were counted in 40 × field (*n* = 6, *p* < 0.05). *Significant difference compared to the PBS-injected group, †Significant difference compared to the BMSC-injected group, *p* < 0.05

To determine whether BDNF-overexpressed BMSCs could differentiate into neurons and promote the repair of neurons in defective spinal cords, we examined the expression of sensory neuron markers, motor neuron markers, and synaptic-related proteins which implied the connections of engrafted BMSC with the neurons around them in the defective spinal cords. Through serial sections of spinal cords and immunofluorescence, we found that the engrafted BDNF-BMSCs could express the sensory neuron marker BRN3A [23], the motor neuron marker ISLET1 [24], and the synaptic-related proteins SNAPSIN1 (SYN) and SNAPTOTAGMIN (SYT) (Fig. 4A–D). The percentage of BRN3A-positive (BRN3A^+^) cells was 35.3 ± 2.3%, ISLET1^+^ cells 17.5 ± 7.6%, SYN^+^ cells 22.3 ± 0.9%, and SYT^+^ cells 18.6 ± 1.5%, respectively. In the whole spinal cords, the mRNA level of these four markers was significantly increased in the BDNF-BMSC-engrafted spinal cords compared to the PBS-injected group and showed a slight but nonsignificant increase compared to the pure BMSC-injected group by RT-qPCR (Fig. 4E–H). Western blot detection showed similar results to RT-qPCR, in that these four neuron-related proteins were significantly increased in BDNF-BMSC- and BMSC-engrafted spinal cords compared to the PBS-injected group. Additionally, the expression of these proteins was significantly increased in BDNF-BMSC-injected spinal cords compared to the PBS-injected group (Fig. 4I–M). The dorsal horn of the spinal cord exposed to the amniotic fluid, which is rich in sensory neurons, is the most severely defective area. As a marker of sensory neurons, BRN3A is necessary for the survival of sensory neurons and the normal growth of sensory axons, and it has been shown to be co-expressed with TrkB, BDNF receptor in the dorsal root ganglion [13, 23]. To verify if BDNF-BMSC transplantation is more beneficial to the recovery of sensory neurons than pure BMSCs in the dorsal spinal cord, the number of BRN3A-positive (BRN3A^+^) cells around the engrafted cells was counted in the sections by immunofluorescence (Fig. 4N–O). The results of statistical analysis showed that the BRN3A^+^ cells were increased significantly in BDNF-BMSC-engrafted spinal cords compared to pure BMSCs-engrafted spinal cords (Fig. 4N, O). Together, these data indicate that intra-amniotic transplantation of BMSCs with BDNF overexpression is more beneficial to the repair of neurons in SBA spinal cords.

### Engrafted BDNF-BMSCs differentiated into skin-like cells near the defective skin

**Fig. 5:**
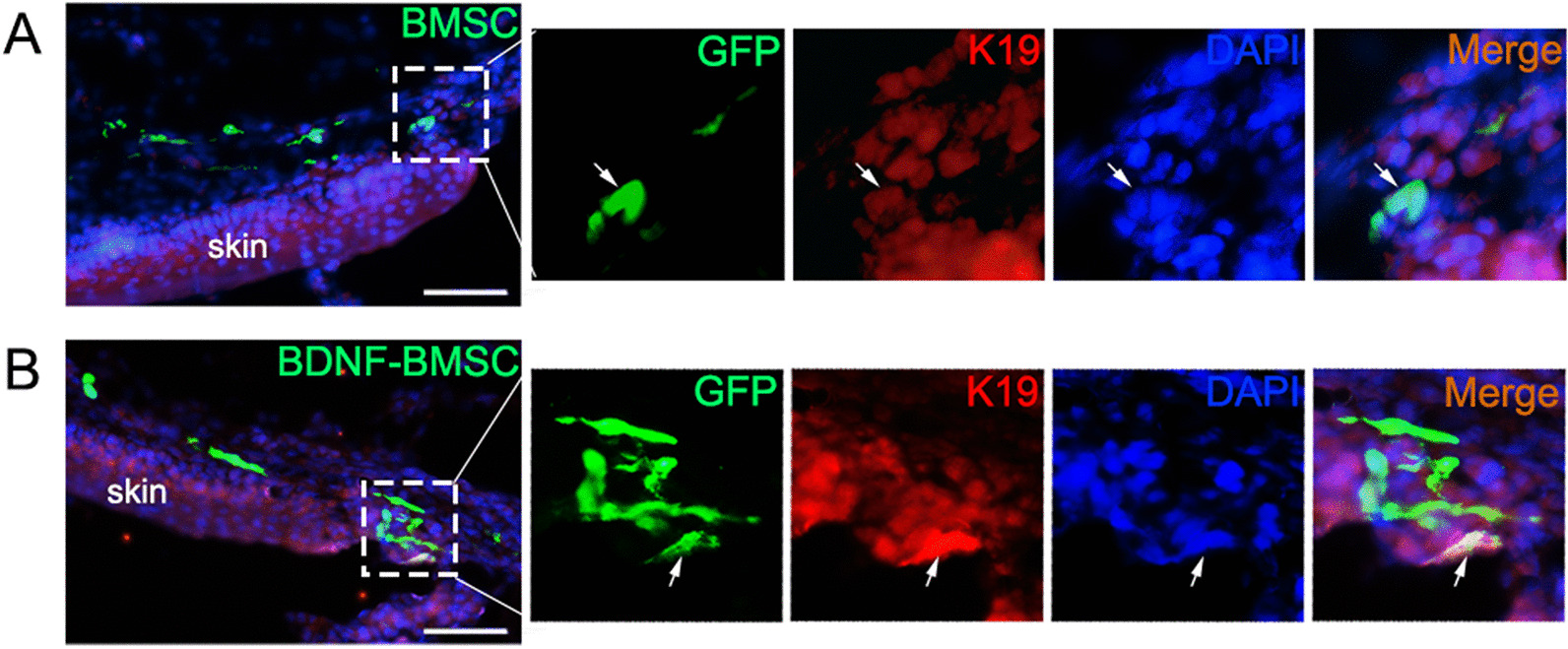
Engrafted BDNF-BMSCs expressed the marker protein K19 of epidermal cells. **A**–**B**. Double immunofluorescence staining of GFP and K19 in frozen sections of rat fetuses with intra-amniotic transplanted BMSC and BDNF-BMSC engrafted. The white box was enlarged on the right side with three color channels. The representative double-positive cells are marked by arrows. Scale bar: 25 μm

Similar to intra-amniotic BMSC transplantation, a component of transplanted BDNF-BMSCs engrafts in the dorsal defective skin of rat fetuses with SBA. Previously, we observed that most of BMSCs engrafted to the edge of the defective skin expressed K19, the marker of epidermal stem cells, which may be helpful for the repair of defective skin. To verify whether genetic modification of BDNF would alter the K19 expression pattern of BMSCs, we investigated the K19 expression of the BDNF-BMSCs that engrafted in the defective skin through frozen section and immunofluorescence staining. We found that BDNF-BMSCs were easily engrafted to the edge of the defective skin and most of these cells also expressed K19 (Fig. 5A, B). BDNF modification did not affect the expression of K19 in engrafted BMSCs; these cells also showed the morphology of epidermal cells. We speculate that these epidermal-like cells derived from BDNF-BMSCs might be involved in the repair of defective skin.

### The amniotic fluid microenvironment was improved by intra-amniotic BDNF-BMSC transplantation

**Fig. 6:**
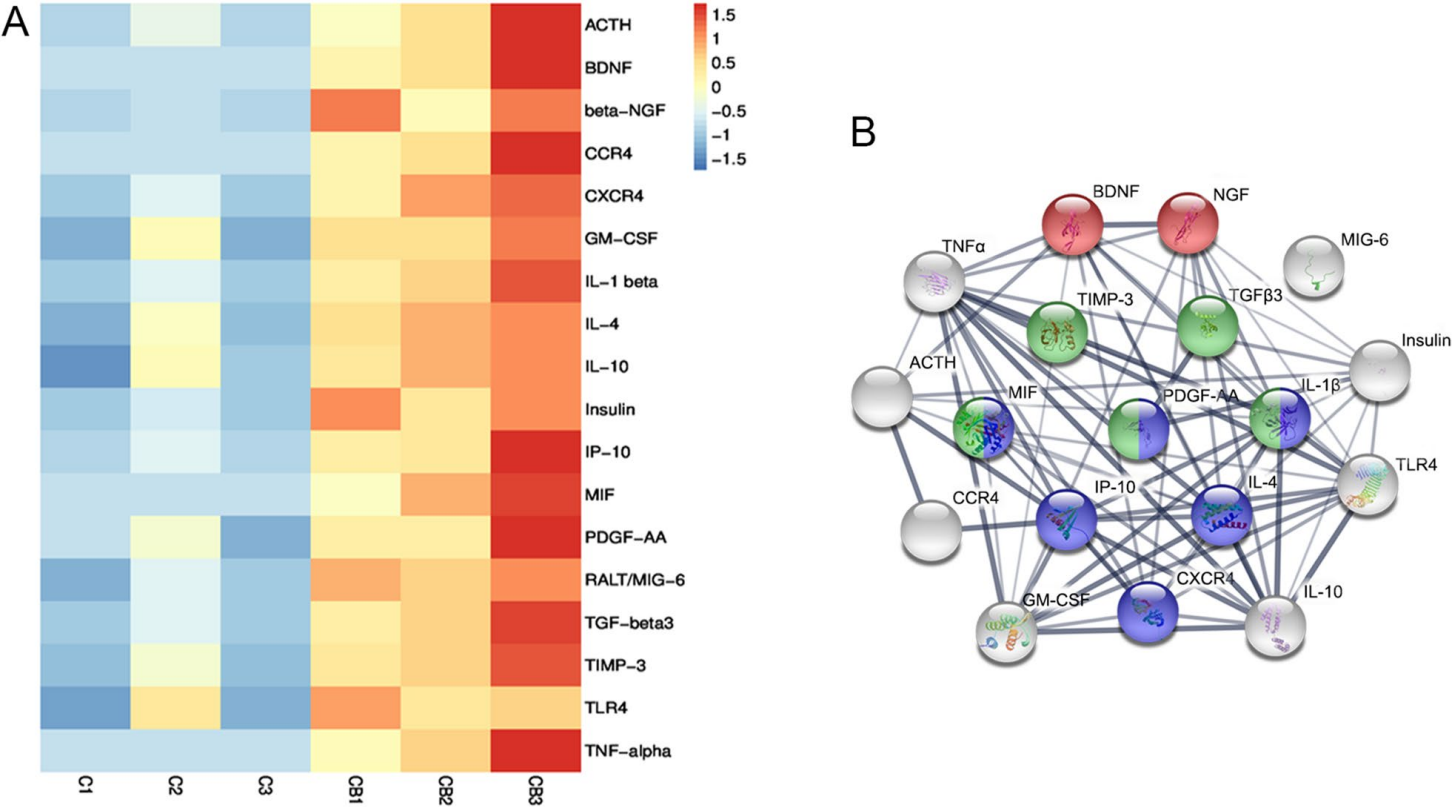
Changes in the amniotic fluid microenvironment after transplantation of BDNF-BMSCs. **A**. Protein array analysis of amniotic fluid with BMSC transplantation (*n* = 3, samples: C1, C2, C3) and BDNF-BMSCs transplantation (*n* = 3, samples: CB1, CB2, CB3). Upregulated proteins (fold change > 3) with *p* < 0.05 are shown in the cluster analysis. **B**. Interaction network of differentially expressed proteins generated by STRING database. The colors of the inside nodes indicate that the proteins come from different biological processes, including negative regulation of neuronal apoptosis (red, GO: 0,043,524), positive regulation of cell migration (blue, GO: 0,030,335), and wound healing (green, GO: 0,042,060), while the proteins that were not involved in the above-mentioned three processes are marked in gray. The thickness of the lines connecting the nodes indicates the strength of data support

To investigate whether BDNF-modified BMSCs could improve the amniotic fluid microenvironment to a greater extent than pure BMSCs, a protein array chip containing 90 factors was used to detect amniotic fluid from E20 of the BDNF-BMSC-transplanted group and pure BMSC-transplanted group. These 90 factors are mainly involved in the processes of nerve regeneration, inflammation, and cell chemotaxis (Additional file 1: Table S1). The results showed that 18 of the factors in amniotic fluid increased significantly (*p* < 0.05 and fold change > 3) in the BDNF-BMSC-transplanted group compared to the pure BMSC-transplanted group (Fig. 6A). Through biological process (GO) analysis, we found that two differentially expressed proteins, the two important neurotrophic factors, BDNF and NGF, were involved in the negative regulation of neuronal apoptosis (red, GO:0,043,524); six cytokines were involved in the positive regulation of cell migration (blue, GO:0,030,335), including MIF, PDGF-AA, IL1β, CXCL10, IL4, and CXCR4; and five cytokines were involved in the processes of wound healing (green, GO:0,042,060), including TIMP3, TGFβ3, MIF, PDGF-AA, and IL1β (Fig. 6B). Considering the interaction between the amniotic fluid microenvironment and the spinal cord environment in SBA embryos/fetuses, the changes in the amniotic fluid microenvironment after BDNF-BMSC transplantation might be conducive to the repair of defective tissues in rat fetuses with SBA.

## Discussion

In the present study, we, for the first time, investigated the expression pattern of BDNF in developing SBA spinal cords and the therapeutic potential of intra-amniotic transplanted BDNF-modified BMSCs. Our results demonstrated that BDNF-BMSC sustained the characteristic of directional migration, could engraft at the lesion area of SBA after intra-amniotic transplantation, increased the expression of BDNF in the defective spinal cords, reduced the apoptosis of spinal cord cells, differentiated into neurons and skin-like cells, reduced the area of defective skin, and improved the amniotic fluid microenvironment. The BDNF-modified BMSCs showed a better effect than pure BMSC in the inhibition of apoptosis and promotion of neural differentiation. These results suggest that intra-amniotic transplanted BDNF-modified BMSCs have an advantage of promoting the recovery of injured neural tissue of SBA fetuses.

Clinically, fetal surgery has been implemented in several centers and achieved promising results. However, postoperative children inevitably present with motor dysfunction [8]. Early repair of abnormal anatomical structure could effectively avoid further damage, while recovery of injured nerves is still an urgent issue that persists in prenatal treatment of children with SBA. As is known, neurotrophins are responsible for improving the survival rate of neurons [25]. BDNF is one such neurotrophin, which promotes the growth and survival of sensory and sympathetic neurons during development [26] and supports the plasticity of the nervous system in different disease models [27, 28]. Indeed, it has been reported that a decreased level of BDNF may contribute to the degeneration of specific neuronal populations and progressive atrophy of neurons in the Alzheimer's disease-affected brain [29]. Through our investigation, we found that the expression of BDNF was significantly decreased in the spinal cord with SBA compared to normal embryo/fetuses in four different stages during development. The diminished level of BDNF might be detrimental to the neural development of fetal rats with SBA and nerve repair after prenatal or postnatal surgical repair.

In the present study, we observed that intra-amniotically transplanted BDNF-BMSCs engrafted into the exposed neural tissue of the SBA spinal cords and the edge of the damaged skin. This engraftment pattern is conducive to the biological role of BDNF in defective tissues. The expression level of BDNF indeed increased in SBA spinal cords after BDNF-BMSCs engraftment. Then, the number of apoptotic cells in the dorsal horns of spinal cords with BDNF-BMSCs engraftment was significantly decreased compared to the PBS-injected group and lower than the BMSC-transplanted group. Changes in the expression of apoptosis-related factors (BCL2, BAX, and CASP3) indicated that intra-amniotic BDNF-BMSCs transplantation was better able to promote the expression of BCL2/BAX and inhibit the expression of CASP3 in SBA spinal cords compared to the BMSCs transplantation (Fig. 3E–G) and Ad-GFP-BDNF delivery that we have tried [13] (see Additional file 2: Fig. S1), likely due to the combined effect of BMSC engraftment and the overexpression of BDNF in the SBA spinal cords. Thus, the intra-amniotic transplantation of BDNF-BMSCs was beneficial to the reduction in apoptosis in SBA spinal cords.

BMSCs could differentiate into neuron-like cells and express corresponding neural markers in different neural tissues or regions [30]. The surrounding cell populations are critical for the differentiation fate of transplanted BDNF-BMSCs. The defective spinal cord and damaged skin were the main sites of transplanted BDNF-BMSCs engraftment. In the spinal cord, most of the BDNF-BMSCs were engrafted at the dorsal horn, an area enriched by sensory neurons [31], and BDNF is required for early sensory neuron development [32, 33]. Therefore, we first evaluated the sensory neuron differentiation potential of the engrafted BDNF-BMSCs. The number of BRN3A^+^ cells around the engrafted BDNF-BMSCs was higher than that around pure BMSCs, and the expression level of BRN3A increased significantly in the spinal cord with engrafted BDNF-BMSCs. This indicates that BDNF-BMSC transplantation has potential value for the recovery of sensory neurons in SBA. We also investigated the effect of BDNF-BMSCs on motor neurons. Only a few cells were found to express ISLET1, the marker of motor neurons, while the expression level of ISLET1 was significantly upregulated in the SBA spinal cord after BDNF-BMSCs engraftment. This might be due to the fact that the anterior horn of the spinal cord is the area where motor neurons develop and not the area of BDNF-BMSCs engraftment, and that overexpressed BDNF might promote survival of motor neurons and upregulated ISLET1 in the defective spinal cords. Synaptic-associated proteins were also detected in the engrafted BDNF-BMSCs and were significantly upregulated in SBA spinal cords with BDNF-BMSCs engraftment. These results collectively suggest that following intra-amniotic transplantation, BDNF-BMSCs promote the restoration of neurons and synapses, which is necessary for neural function recovery.

In addition, it has been reported that BMSCs have the potential to differentiate into a variety of skin cell types and contribute to skin regeneration [34–37]. We previously demonstrated that BMSCs also have the potential to transdifferentiate into epidermal cells, because the percentage of K19-positive BMSCs was higher when engrafted at defective skin than when cultured in vitro [18]. In the current study, at the edge of the damaged skin, we found that the engrafted BDNF-BMSCs sustain the differentiation potential of BMSCs to K19^+^ skin cells. BDNF belongs to the neurotrophins family, which also fulfills multiple regulatory functions outside the nervous system. BDNF has effects on skin cell fate (survival, apoptosis, proliferation, and differentiation) which are likely to strongly depend on signaling pathways that are activated by the binding to specific receptors (TrkB and P75NTR) [38, 39]. It has been reported that keratinocytes could express BDNF and its receptors; BDNF-lacking transgenic mice showed a significantly reduced number of proliferating epidermal keratinocytes [40, 41]. It is speculated that BDNF-BMSC may involve in SBA skin repair by differentiating into skin-like cells, secreting BDNF to affect the survival or proliferation of skin cells. Further efforts are required to fully understand molecular mechanisms underlying the involvement of BDNF-BMSCs in skin repair of SBA.

After intra-amniotic BMSCs-BDNF transplantation, the composition of amniotic fluid changed compared to that following BMSC transplantation. According to the differentially expressed cytokines, we suggest that BMSCs overexpressing BDNF saved injured neurons in SBA spinal cords and promoted the expression of other growth factors in amniotic fluid. This might be due to the overexpression of BDNF, which increases BMSC-secreted neurotrophic factors into the amniotic fluid, reduces apoptosis, increases the survival of spinal cord nerve cells, and further promotes the repair of injury. Among the upregulated neurotrophic factors, NGF is a well-studied factor that has shown therapeutic value for diseases of the peripheral nervous system (PNS) and central nervous system (CNS), as well as on the visual system and cutaneous wound healing [42]. Many reports have suggested that TGFβ3 has potential applications in wound healing [43], cartilage repair [44], and autoimmune diseases [45] by modulating cellular proliferation and migration. The upregulation of anti-inflammatory IL-4 could regulate cellular survival, proliferation, and branching in the PNS and CNS and promotes peripheral axon regeneration, while upregulation of IL-10 is implicated in cell survival (CNS and PNS) and may promote recovery after spinal cord injury [46]. It has been reported that neurotrophin-induced neurite outgrowth is dose-dependently modulated by pro- and anti-inflammatory cytokines [46]. These upregulated cytokines related to the regulation of the apoptotic process and the generation of neurons may explain the reduction in apoptosis and increase in BRN3A^+^ neurons in SBA spinal cords; however, the specific effects of these cytokines on the developing SBA fetuses still need to be further studied.

Collectively, BDNF-modified BMSCs have shown certain advantages for intra-amniotic SBA treatment in this study. Due to the limitation of the animal model, it is difficult to investigate the long-term recovery of neural function of fetal rats after birth. Nerve repair of SBA takes a long time, and large animal models may be needed to identify the long-term effects of intra-amniotic stem cell transplantation. Whether intra-amniotic genetically modified stem cell transplantation can be used as an independent minimally invasive method or as an adjunct to the existing prenatal surgical treatment in the clinic for SBA remains to be further studied and evaluated.

## Conclusion

In summary, intra-amniotic transplantation of BDNF-modified BMSCs showed therapeutic potential for SBA. This method of intra-amniotic stem cell transplantation provides possible access to dysplastic neural and skin tissue as a result of lesion-specific engraftment, thereby improving the SBA microenvironment. The resulting specific cell differentiation participates in lesion repair and nerve reconstruction in early pregnancy. Although this treatment requires further exploration, it may offer novel ways to improve the regeneration and plasticity of affected tissue to improve outcomes for SBA and other congenital malformations.

## Supplementary Information

**Additional file 1**: **Table S1**. The fold changes and *P* values for all proteins in protein array.

**Additional file 2**: **Fig. S1**. Effects of intra-amniotic injection of Ad-GFP-BDNF, BMSC, and BDNF-BMSC on apoptosis and synaptogenesis of SBA spinal cords.

## Data Availability

All data generated or analyzed during this study are included in this published article and its supplementary files.

## Abbreviations

atRA: All-trans-retinoic acid
BAX: Pro-apoptotic Bcl 2-associated X
BCL2: Anti-apoptotic B cell lymphoma 2
BDNF: Brain-derived neurotrophic factor
BMSC: Bone marrow mesenchymal stem cells
BRN3A: POU class 4 homeobox 1
CASP3: Caspase-3
CNS: Central nervous system
GAPDH: Glyceraldehyde-3-phosphate dehydrogenase
GFP: Green fluorescent protein
HRP: Horseradish peroxidase
IL-10: Interleukin 10
IL-4: Interleukin 4
ISLET1: LIM-homeodomain transcription factor 1
K19: Cytokeratin-19
MMC: Myelomeningocele
NGF: Nerve growth factor
PBS: Phosphate-buffered saline
PNS: Peripheral nervous system
SBA: Spina bifida aperta
SYN: Synapsin1
SYT: Synaptotagmin1
TGFβ3: Transforming growth factor-β 3
TUNEL: TdT-mediated dUTP nick-end labeling
