# Supplementary material for: Intra-amniotic transplantation of brain-derived neurotrophic factor-modified mesenchymal stem cells treatment for rat fetuses with spina bifida aperta
Source: Stem Cell Res Ther. 2022 Aug 13;13:413. doi: 10.1186/s13287-022-03105-6 (PMC9375302; doi:10.1186/s13287-022-03105-6)
Supplement: Supplementary file 1 — Additional file 1: Table S1. The fold changes and P values for all proteins in protein array. [file 13287_2022_3105_MOESM1_ESM.docx]

**Supplementary table**

| **Table S1. The fold changes and *P* values for all proteins in protein array.** | | | |
| --- | --- | --- | --- |
| NO. | **Name of the protein** | **Fold change** | **P value** |
| 1 | **Activin A** | 85.67 | 0.12 |
| 2 | **ACTH** | 8.85 | 0.03 |
| 3 | **ADFP** | 101.67 | 0.08 |
| 4 | **Adiponectin/Acrp30** | 0.74 | 0.17 |
| 5 | **AMPK alpha 1** | 0.06 | 0.05 |
| 6 | **B7-1/CD80** | 38 | 0.19 |
| 7 | **BDNF** | 119.67 | 0.04 |
| 8 | **beta-Catenin** | 37.33 | 0.19 |
| 9 | **basic-FGF** | 37 | 0.19 |
| 10 | **beta-NGF** | 33.86 | 0.03 |
| 11 | **CCR4** | 76.33 | 0.03 |
| 12 | **CD106** | 1.08 | 0.38 |
| 13 | **CINC-2 alpha/beta** | 0.34 | 0.02 |
| 14 | **CINC-3** | 0.38 | 0.02 |
| 15 | **CNTF** | 51 | 0.19 |
| 16 | **CNTF R alpha** | 17.67 | 0.19 |
| 17 | **CSK** | 38 | 0.19 |
| 18 | **CXCR4** | 11.9 | 0.01 |
| 19 | **EGFR** | 38.33 | 0.19 |
| 20 | **EG-VEGF/PK1** | 0.47 | 0.2 |
| 21 | **E-Selectin** | 109.33 | 0.07 |
| 22 | **FADD** | 36.33 | 0.19 |
| 23 | **Fas/TNFRSF6** | 0.58 | 0.28 |
| 24 | **Fas Ligand/TNFSF6** | 32 | 0.19 |
| 25 | **FGF-BP** | 0.18 | 0.19 |
| 26 | **Follostatin-like -1(FSL1)** | 1101 | 0.16 |
| 27 | **Fractalkine** | 18.33 | 0.19 |
| 28 | **GFR alpha-1** | 52.67 | 0.09 |
| 29 | **GFR alpha-2** | 229.67 | 0.05 |
| 30 | **GM-CSF** | 4.51 | 0.02 |
| 31 | **Growth Hormone** | 0.33 | 0.20 |
| 32 | **Growth Hormone R** | 1.37 | 0.12 |
| 33 | **Hepassocin** | 0.27 | 0.16 |
| 34 | **ICAM-1/CD54** | 174.67 | 0.06 |
| 35 | **ICK** | 0.29 | 0.02 |
| 36 | **Insulin Degrading Enzyme** | 2.71 | 0.08 |
| 37 | **IFN-gamma** | 38.33 | 0.19 |
| 38 | **IL-1 alpha** | 130.67 | 0.07 |
| 39 | **IL-1 beta** | 10.46 | 0.01 |
| 40 | **IL-1 R6/IL-1 R rp2** | 175 | 0.07 |
| 41 | **IL-2** | 0.8 | 0.38 |
| 42 | **IL-3** | 35 | 0.16 |
| 43 | **IL-4** | 4.52 | 0.01 |
| 44 | **IL-5** | 0.4 | 0.04 |
| 45 | **IL-6** | 1.78 | 0.27 |
| 46 | **IL-10** | 3.38 | 0.02 |
| 47 | **IL-12/IL-23 p40** | 0.24 | 0.03 |
| 48 | **IL-13** | 102 | 0.07 |
| 49 | **Integrin alpha M beta 2** | 0.52 | 0.09 |
| 50 | **Insulin** | 12.68 | 0.00 |
| 51 | **IP-10** | 14.09 | 0.01 |
| 52 | **Leptin (OB)** | 80 | 0.14 |
| 53 | **LIX** | 33.67 | 0.19 |
| 54 | **L-Selectin/CD62L** | 58.67 | 0.19 |
| 55 | **MCP-1** | 2.1 | 0.02 |
| 56 | **MDC** | 0.26 | 0.08 |
| 57 | **MIF** | 185.33 | 0.04 |
| 58 | **MIP-1 alpha** | 127.33 | 0.07 |
| 59 | **MIP-2** | 1.81 | 0.09 |
| 60 | **MIP-3 alpha** | 77 | 0.13 |
| 61 | **MMP-2** | 2.24 | 0.02 |
| 62 | **MMP-8** | 48.67 | 0.08 |
| 63 | **MMP-13** | 122 | 0.06 |
| 64 | **MuSK** | 0.37 | 0.28 |
| 65 | **Neuropilin-2** | 0.71 | 0.21 |
| 66 | **NGFR** | 109.33 | 0.09 |
| 67 | **Orexin A** | 35.33 | 0.19 |
| 68 | **Osteopontin/SPP1** | 104.33 | 0.09 |
| 69 | **PDGF-AA** | 3.75 | 0.03 |
| 70 | **Prolactin R** | 0.31 | 0.04 |
| 71 | **RAGE** | 29 | 0.19 |
| 72 | **RALT/MIG-6** | 6.24 | 0.00 |
| 73 | **RELM beta** | 0.38 | 0.05 |
| 74 | **Resistin** | 0.15 | 0.21 |
| 75 | **TAL1A** | 55 | 0.17 |
| 76 | **TGF-beta1** | 38 | 0.19 |
| 77 | **TGF-beta2** | 37 | 0.19 |
| 78 | **TGF-beta3** | 12.54 | 0.01 |
| 79 | **Thrombospondin** | 1.87 | 0.14 |
| 80 | **TIE-2** | 63.33 | 0.19 |
| 81 | **TIMP-1** | 39.33 | 0.19 |
| 82 | **TIMP-2** | 2.01 | 0.01 |
| 83 | **TIMP-3** | 5.48 | 0.01 |
| 84 | **TLR4** | 3.09 | 0.04 |
| 85 | **TNF-alpha** | 276.67 | 0.04 |
| 86 | **TRAIL** | 0.56 | 0.15 |
| 87 | **TROY** | 1.15 | 0.15 |
| 88 | **Ubiquitin** | 0.55 | 0.03 |
| 89 | **VEGF** | 0.53 | 0.12 |
| 90 | **VEGF-C** | 36 | 0.19 |
