# Supplementary material for: Intra-amniotic transplantation of brain-derived neurotrophic factor-modified mesenchymal stem cells treatment for rat fetuses with spina bifida aperta
Source: Stem Cell Res Ther. 2022 Aug 13;13:413. doi: 10.1186/s13287-022-03105-6 (PMC9375302; doi:10.1186/s13287-022-03105-6)
Supplement: Supplementary file 2 — Additional file 2: Fig. S1. Effects of intra-amniotic injection of Ad-GFP-BDNF, BMSC, and BDNF-BMSC on apoptosis and synaptogenesis of SBA spinal cords. [file 13287_2022_3105_MOESM2_ESM.docx]

**Supplementary figure**


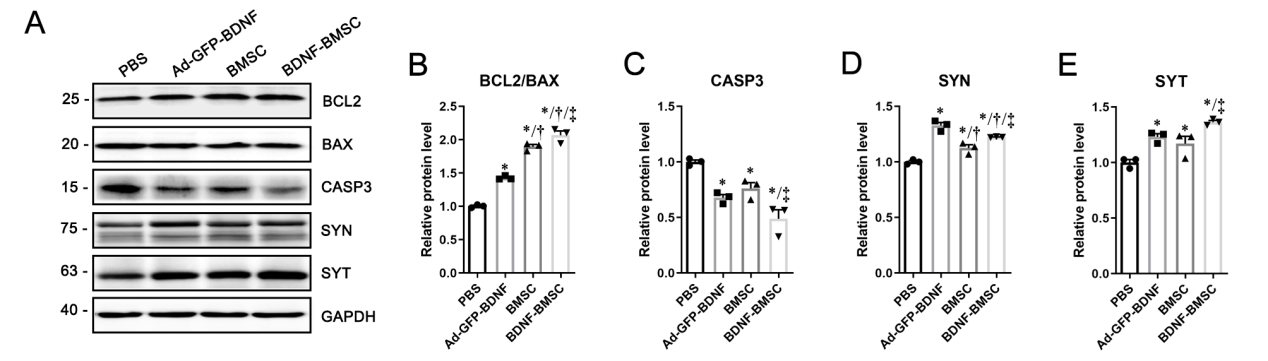


**Figure S1. Effects of intra-amniotic injection of Ad-GFP-BDNF, BMSC and BDNF-BMSC on apoptosis and synaptogenesis of SBA spinal cords. A**: Protein expressions of BCL2, BAX, CASP3, SYN, SYT in spinal cords from SBA fetuses after intra-amniotic injection of PBS, Ad-GFP-BDNF, BMSCs and BDNF-BMSCs were detected by western blot. These proteins were separated using sodium dodecyl sulfate polyacrylamide gel electrophoresis (SDS-PAGE) and transferred to polyvinylidene difluoride (PVDF) membranes. The membranes were blocked with skim milk at room temperature for 1 hour, then washed with TBST and incubated with specific primary antibodys at 4°C overnight. The next day, the membranes were washed with TBST and incubated with corresponding horseradish peroxidase (HRP)-conjugated IgG at room temperature for 1 hour. ECL-Plus kit (Merck Millipore, Darmstadt, Germany) was used to detect the signals. **B-E**: Quantification of relative protein levels of BCL2/BAX, CASP3, SYN, SYT determined from immunoblots in figure A. *Significant difference compare to PBS-injected group, †Significant difference compare to Ad-GFP-BDNF-injected group, ‡Significant difference compare to BMSC-injected group, p<0.05.
